# Enzyme Activities Shape Malting Quality Standards

**DOI:** 10.1002/fsn3.4702

**Published:** 2024-12-19

**Authors:** Kangfeng Cai, Xiaojian Wu, Wenhao Yue, Lei Liu, Xiujuan Song, Fangying Ge, Qiuyu Wang, Junmei Wang

**Affiliations:** ^1^ Institute of Crop and Nuclear Technology Utilization Zhejiang Academy of Agricultural Sciences Hangzhou China; ^2^ College of Agriculture Yangtze University Jingzhou China; ^3^ College of Advanced Agricultural Sciences Zhejiang Agriculture and Forestry University Hangzhou China; ^4^ College of Life Sciences Zhejiang Normal University Jinhua China

**Keywords:** barley, correlation, enzyme activity, gene expression, malting quality

## Abstract

Malting quality of barley is a complex characteristic, which is influenced by a combination of interacting traits that are regulated by various genetic and environmental factors. The activities of various enzymes play pivotal roles in determining the malting quality, as they drive the biochemical processes responsible for converting barley saccharides and proteins into fermentable sugars and amino acids during the malting process. In this study, 14 malting barley cultivars were used to investigate the relationship between enzyme activities and malting quality traits. The results revealed a significant correlation between α‐amylase activity and malt extract (MEX), viscosity (VIS), free α‐amino nitrogen (FAN), and Kolbach index (KI). In contrast, β‐amylase activity exhibited a significant correlation solely with diastatic power (DP). β‐glucanase activity was significantly correlated with FAN and KI. The elevated expression levels of both *HvBmy1* and *HvBmy2* contributed to high DP, and the activation of α‐amylase genes (*HvAmy1* and *HvAmy2*) and β‐glucanase genes (*HvGlb1* and *HvGlb2*) played a crucial role in producing high FAN and KI. These results enhance our understanding of the relations between enzyme activity and malting quality traits and thereby may facilitate further breeding for malt barley cultivars.

## Graphical abstract

Overall, α‐amylase activity, β‐amylase activity, and β‐glucanase activity in malt were significantly correlated with malting qualities. The expression of genes encoding α‐amylase, β‐amylase, and β‐glucanase were remarkably increased and exhibited similar patterns during germination in cultivars with elite malting quality traits.

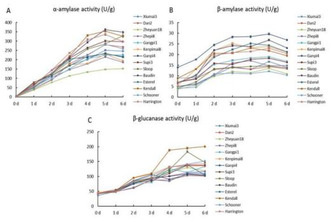

## 1 Introduction

Malt constitutes the primary constituent used in the brewing industry. Malt is derived from barley ( *Hordeum vulgare* L.) grains through controlled germination, leading to the physical and biochemical alteration of the barley endosperm (Yousif and Evan Evans 2020). The malting process can be categorized into three distinct stages: steeping, germination, and kilning (Kumar, Chaturvedi, and Singh 2023). Hydrolytic enzymes are synthesized and/or released to degrade starch, cell wall nonstarch polysaccharides, proteins, and lipids during germination, which is crucial for endosperm carbohydrates and protein modification, and utilization during malt mashing (Rani and Whitcomb 2025; Yousif and Evan Evans 2020).

Malting quality of barley is a complex characteristic, which is influenced by not only genetics, environment, and their interactions, but also the technical operation of the malting process (Leisova‐Svobodova et al. 2024). Malting quality traits encompass malt extract (MEX), wort β‐glucan (BG) content, wort viscosity (VIS), Kolbach index (KI), free α‐amino nitrogen (FAN), soluble protein (SP), diastatic power (DP), α‐amylase (EC 3.2.1.1) activity, β‐amylase (EC 3.2.1.2) activity, friability, β‐glucanase (EC 3.2.1.73) activity, and fermentability (Fox et al. 2003). The brewing industry requires malt with high fermentable sugar and malt extract levels, low wort viscosity, high DP, optimal protein content, and low BG content for good malting quality (Bamforth 2009). The main objectives in malting include synthesis of various enzymes within the grain (e.g., α‐amylase, β‐amylase, and β‐glucanase), enzymatic breakdown of barley endosperm cell walls (predominantly β‐glucan), and cellular contents (a portion of the endosperm protein), as well as the development of desirable malt color and flavor (Briggs 1998; Laitila et al. 2007). MEX is an important indicator of malting quality and is influenced by grain development (Fox et al. 2003). DP is the total activity of malt enzymes that hydrolyze starch to fermentable sugars, which leads to elevated FAN levels and involves α‐amylase and β‐amylase (Cu et al. 2016). The characteristics and properties of them significantly impact the fermentability of wort (Evans et al. 2005). And β‐amylase activity was found to be a better predictor of DP compared with α‐amylase (Georg‐Kraemer, Mundstock, and Cavalli‐Molina 2001). The degradation of grain protein serves as a crucial source of amino acids, which is vital for yeast growth during fermentation; however, an excessive amount of grain protein results in a decrease in MEX (Qi et al. 2005). BG is the major component of endosperm cell wall, and cell wall contains around 70% BG (Kuusela et al. 2004). During germination, β‐glucanase is synthesized and catalyzes the breakdown of BG (Kuusela et al. 2004). Insufficient degradation of cell wall leading to a high wort BG content may impede enzyme diffusion in germinated grains and result in a reduction of MEX (Bamforth 2003). The presence of residual BG in malt and solubilization of high molecular weight BG can also result in high VIS, thereby causing filtration problems (Bamforth 2003; Lai et al. 2004). And proteinases degrade large and typically insoluble storage proteins into soluble proteins, peptides, and amino acids (Simpson 2001). KI is a measure of the degree of protein degradation in malt, calculated as the ratio of soluble nitrogen content to total nitrogen content (Liu et al. 2021).

The objective of this study was to elucidate the relationship between saccharide‐hydrolyzing enzymes such as α‐amylase, β‐amylase and β‐glucanase, and malting quality traits, and also to uncover the universal regulatory mechanisms of these genes underpinning cultivars with elite malting quality traits.

## 2 Materials and Methods

### 2.1 Plant Growth and Sampling

A total of 14 malt barley cultivars were used in this study, which are widely recognized and commonly employed (Table 1). All cultivars were grown in mid‐November 2015 in Hangzhou, Zhejiang Province (HZ, 30°25′ N, 120°17′ E), which has a subtropical monsoon climate. The preceding crop was rice and the soil type was silt‐loam with medium fertility. The experiments were conducted utilizing a randomized complete block design with three replicates. The fertilization, disease, and pest control were carried out as described in previous research (Wang et al. 2018). Manual weeding was carried out as required. The average high/low temperatures from November 2015 to May 2016 were 17°C/11°C, 11°C/5°C, 7°C/2°C, 13°C/3°C, 17°C/8°C, 22°C/14°C, and 26°C/18°C, respectively. The grains were harvested and subsequently stored at −4°C for further analyses.

**TABLE 1:** Varieties used in this study.

Variety	Source	Variety	Source
Xiumai3	China	Supi3	China
Dan2	China	Sloop	Australia
Zheyuan18	China	Baudin	Australia
Zhepi8	China	Esterel	France
Gangpi1	China	Kendall	Canada
Kengpimai8	China	Schooner	Australia
Ganpi4	China	Harrington	Canada

### 2.2 Micro‐Malting and Measurements

The barley grains were sieved using a 2.2‐mm mesh, and the retained grains on the sieve were used for micromalting. 200 g grains of each cultivar were subjected to micromalting in a Joe White Micro‐malting System Apparatus (Adelaide, Australia), following the specified procedure: steeping (16°C, 6 h), air‐rest (16°C, 14 h), steeping (16°C, 8 h), air‐rest (16°C, 14 h), steeping (16°C, 4 h); germination at 15°C for 96 h; kilning at 65°C for 24 h. The malt samples were collected on a daily basis starting from the initiation of micromalting (0, 1, 2, 3, 4, 5, 6, and 7 days). The malt samples were partitioned into four parts (endosperm, scutellum, root, and shoot), snap‐freezed, and then stored at −80°C for further analysis.

### 2.3 Malting Quality Traits Determination

The malting quality traits, including MEX, KI, VIS, and DP, were determined as described (Wang et al. 2015). The activities of α‐amylase, β‐amylase, and β‐glucanase were determined using enzyme activity assay kits (Megazyme, Ireland) following manufacturer's instructions.

### 2.4 RNA Extraction and qRT‐PCR

Total RNA was extracted using RNeasy Plant Mini Kit (Qiagen, Germany) according to its protocol, and then reverse transcribed to cDNA using SuperScript III First‐Strand Synthesis SuperMix (Invitrogen, USA). Quantitative real‐time PCR (qRT‐PCR) was performed to determine the relative transcript level of six malting quality‐related genes (Table 2) using Power SYBR Green PCR Master Mix kit (Applied Biosystems, USA). Primers used are listed in Table 2. The relative expression levels of genes in endosperm, scutellum, and shoot at 1 day of germination were normalized to 1, while that in root at 2 days of germination were normalized to 1.

**TABLE 2:** Primers used for qRT‐PCR.

Gene	Genbank accession	Primer sequences (5′ to 3′, forward/reverse)	Product (bp)	Annealing (°C)
*Barley 18S*	AY552749.1	CGCTCTGGATACATTAGCATGG	162	60
		GCTTTCGCAGTTGTTCGTCTTTCA		
*HvAmy1*	M17128.1	GTCTGCACTGATCCGTCATTCGAT	140	60
		CTACAGTCGTGTGAGCAATTCGTA		
*HvAmy2*	FN179390	CCTCATTCCTGAAGGCTTCAAAGT	97	60
		AATTTGTAGAGCCGCTCCGTTAAT		
*HvBmy1*	FN179393	TGCCGTCCAGATGTATGCCGATTA	106	60
		AGCTGGGCCAAGTCCTACTTCAAT		
*HvBmy2*	FN179394	AGCGCACCAGAAGAACTAGTCCAA	135	60
		TTTCGGCCTCGCATTCCTGAGTAT		
*HvGlb1*	X56775	ACGCCGTACGTATGCGCACATTAT	158	60
		GCGTTTGCATATGCTTCCCTTCCA		
*HvGlb2*	AK251293	CCTCTTAATTACCTCCTCTTTCCA	153	60
		CATTTACGGTTGCTACGTTATGAC		

### 2.5 Statistical Analysis

Analysis of variance (ANOVA) was carried out among barley genotypes and followed by the least significant difference (LSD) multiple range test (*p* < 0.05), using SPSS 20.0 (IBM, USA).

## 3 Results

### 3.1 Statistical Analysis of Malting Quality Traits

**FIGURE 1:**
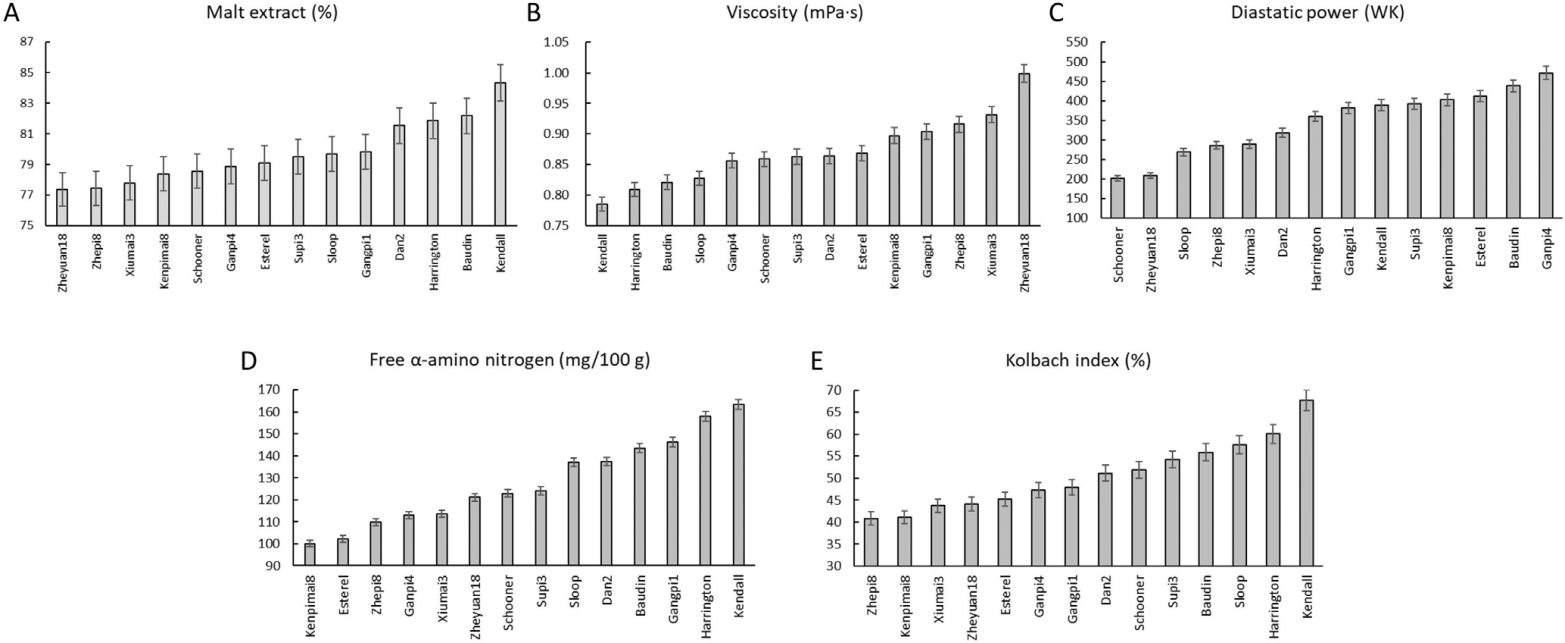
Malt extract (A), viscosity (B), diastatic power (C), free α‐amino nitrogen (D), and Kolbach index (E) of malt from 14 cultivars.

The 14 cultivars exhibited significant differences in malting quality traits: MEX, VIS, FAN, KI, and DP (Table 3). DP varied from 202.28 WK to 471.27 WK and showed the highest degree of variation among 14 cultivars with a coefficient of variation (CV) of 24.16%. FAN, KI, and VIS varied from 100.1 mg/100 g to 163.41 mg/100 g, 40.84% to 67.75%, and 0.79 mPa·s to 1.00 mPa·s, with the CV of 15.60%, 15.48%, and 5.75%, respectively (Table 3). MEX displayed the lowest CV of 2.56% and ranged from 77.36% to 84.33% (Table 3). MEX of Kendall was the highest among 14 cultivars, reaching 84.33% (Figure 1A). MEX of Dan2, Harrington, Baudin were also exceeded 81%. Kendall displayed the lowest VIS of 0.79 mPa·s, followed by Harrington of 0.81 mPa·s. Zheyuan18 displayed the highest VIS of 1.00 mPa·s (Figure 1B). DP of Schooner and Zheyuan18 were lower than 210 WK, while that of the others were higher than 250 WK (Figure 1C). DP of Ganpi4 reached a peak of 471.27 WK. Kendall exhibited the highest FAN content and KI, reaching 163.41 mg/100 g and 67.75%, followed by Harrington with 158.01 mg/100 g and 60.12%, respectively (Figure 1D,E).

**TABLE 3:** Statistical analysis of malting quality traits of 14 varieties.

Trait	*F*	Minimum	Maximum	Mean	SD	CV%
MEX (%)	1.54	77.36	84.33	79.74	2.04	2.56
VIS (mPa·s)	9.01**	0.79	1.00	0.87	0.05	5.75
FAN (mg/100 g)	56.01**	100.1	163.41	128.07	19.98	15.60
KI (%)	55.26**	40.84	67.75	50.63	7.84	15.48
DP (WK)	130.38**	202.28	471.27	344.44	83.23	24.16

Abbreviations: DP, diastatic power; FAN, free α‐amino nitrogen; KI, Kolbach index; MEX, malt extract; VIS, viscosity.

** Significance at *p* < 0.01.

### 3.2 Dynamics of Enzyme Activity

**FIGURE 2:**
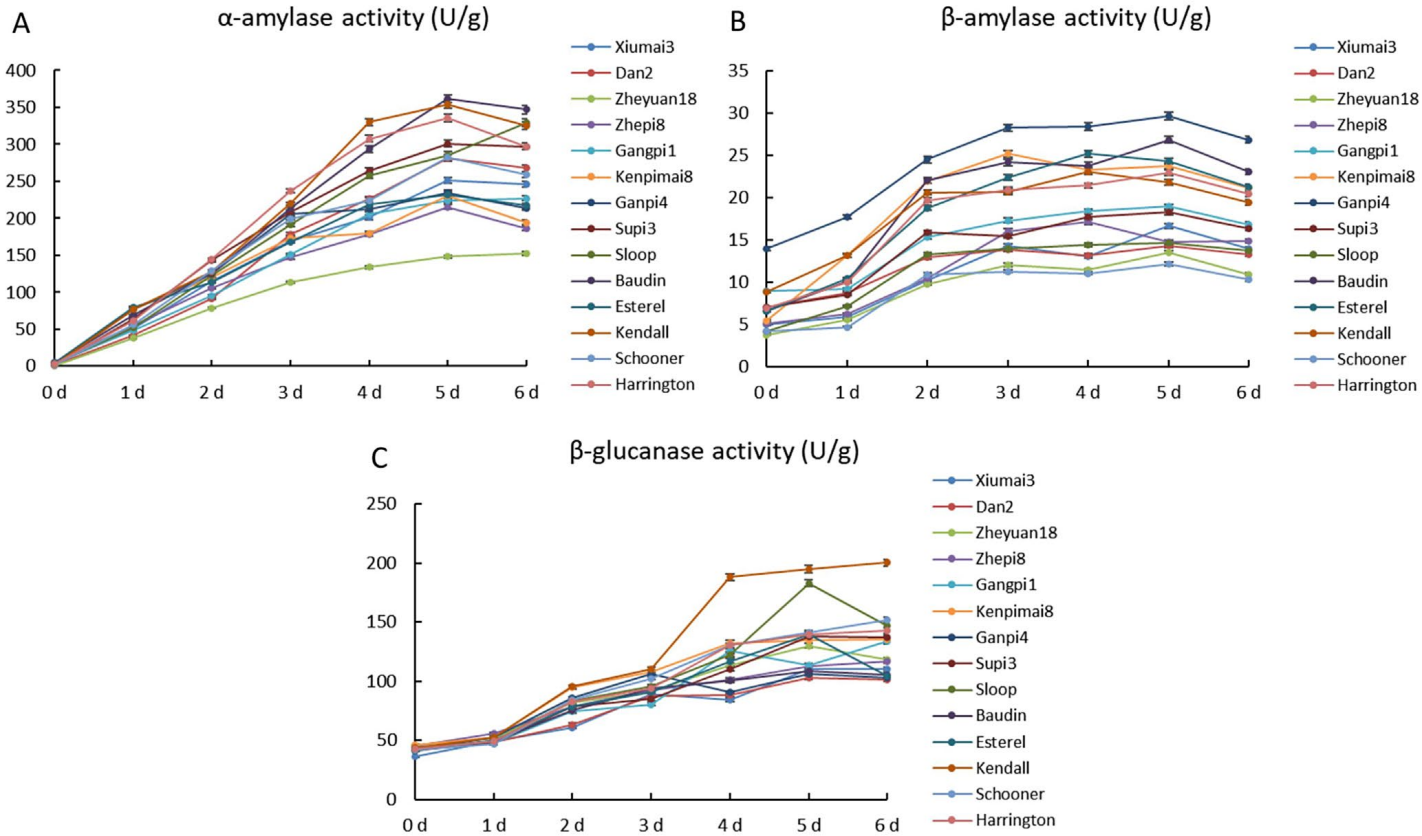
Dynamics of α‐amylase activity (A), β‐amylase activity (B), and β‐glucanase activity (C) of 14 cultivars after germination.

The dynamics of hydrolytic enzyme activity after germination was investigated. The activity of α‐amylase, β‐amylase, and β‐glucanase of 14 cultivars generally increased rapidly during germination (Figure 2). α‐amylase activity of tested cultivars increased and peaked at 5 days of germination and subsequently slightly decreased, except that of Zheyuan18, Gnagpi1, and Sloop, which peaked at the end of germination procedure (Figure 2A). α‐amylase activity of Kendall, Harrington, and Baudin exhibited the highest rate of increase and maximum value. β‐amylase activity of all cultivars displayed a rapid increase during the first 3 days and then a slight increase until 5 days. β‐amylase activity of Ganpi4 was consistently higher than that of other cultivars during the whole germination period (Figure 2B). β‐glucanase activity of all cultivars increased during the germination period and peaked at 5–6 days. Notably, Kendall exhibited remarkably higher β‐glucanase activity after 4 days of germination compared with other cultivars (Figure 2C).

### 3.3 Correlation Among Enzyme Activity and Malting Quality Traits

Correlation among hydrolytic enzymes and malt quality traits was investigated (Table 4). β‐glucanase activity showed a significant and positive correlation with FAN and KI. It also exhibited a positive correlation with MEX and a negative correlation with VIS, although the correlation did not reach statistical significance. α‐amylase activity was significantly and negatively correlated with VIS, while significantly and positively correlated with MEX, FAN, and KI. β‐amylase activity displayed significant and positive correlation with DP. Among malt quality traits, MEX showed a significant and negative correlation with VIS, while a significant and positive correlation with FAN and KI. VIS exhibited a significant and negative correlation with FAN and KI. FAN was significantly and positively correlated with KI.

**TABLE 4:** Correlation between the three enzymes and five malting quality traits.

	β‐glucanase activity	α‐amylase activity	β‐amylase activity	MEX	VIS	FAN	KI
α‐amylase activity	0.4						
β‐amylase activity	−0.02	0.11					
MEX	0.47	0.76**	0.31				
VIS	−0.46	−0.83**	−0.38	−0.81**			
FAN	0.54*	0.69**	−0.06	0.84**	−0.59*		
KI	0.67**	0.86**	0.04	0.87**	−0.82**	0.86**	
DP	−0.12	0.22	0.86**	0.42	−0.47	0.06	0.15

Abbreviations: DP, diastatic power; FAN, free α‐amino nitrogen; KI, Kolbach index; MEX, malt extract; VIS, viscosity.

* Significance at *p* < 0.05 and *p* < 0.01, respectively.

** Significance at *p* < 0.05 and *p* < 0.01, respectively.

### 3.4 The Expression Profiling of Malting Quality‐Related Genes

Generally, Kendall and Harrington exhibited high MEX, FAN, and KI, low VIS as well as moderate DP, while Ganpi4 exhibited the highest DP (Figure 1). On the other hand, MEX, FAN, KI, and VIS were significantly correlated with α‐amylase activity; FAN and KI were significantly correlated with β‐glucanase activity; and DP was significantly correlated with β‐amylase activity (Table 4). Therefore, Kendall and Harrington were used for expression analysis of α‐amylase (*HvAmy1* and *HvAmy2*) and β‐glucanase genes (*HvGlb1* and *HvGlb2*), while Kendall and Ganpi4 were used for expression analysis of β‐amylase genes (*HvBmy1* and *HvBmy2*).

**FIGURE 3:**
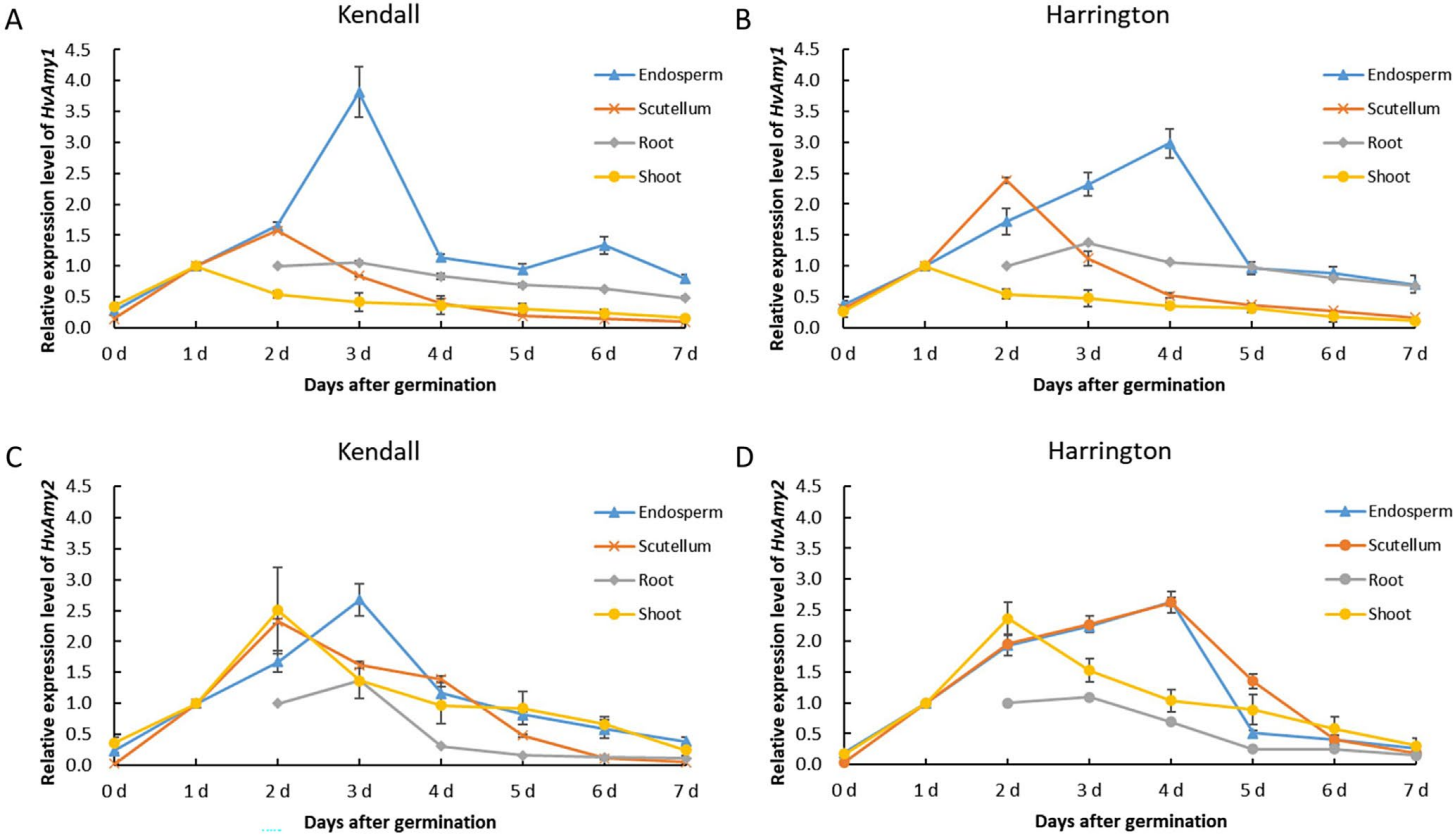
Relative expression level of *HvAmy* genes in four tissues of Kendall and Harrington. (A) Relative expression of *HvAmy1* in Kendall; (B) relative expression of *HvAmy1* in Harrington; (C) relative expression of *HvAmy2* in Kendall; (D) relative expression of *HvAmy2* in Harrington.

The expression levels of *HvAmy1* in endosperm increased, and peaked at 3 days and 4 days in Kendall and Harrington, respectively, and decreased subsequently (Figure 3A,B). In scutellum, the highest expression levels of *HvAmy1* were observed at 2 days for both cultivars, and the expression level in Harrington was slightly higher than that in Kendall. In root and shoot, the expression levels of *HvAmy1* were relatively low. Likewise, the expression levels of *HvAmy2* in endosperm summited at 3 days and 4 days in Kendall and Harrington, respectively, followed by a rapid decline thereafter (Figure 3C,D). The expression of *HvAmy2* in the scutellum of Kendall reached its peak at 2 days, whereas in Harrington, the highest expression level was observed at 4 days. The expression levels of *HvAmy2* in shoots also increased and peaked at 2 days in both cultivars.

**FIGURE 4:**
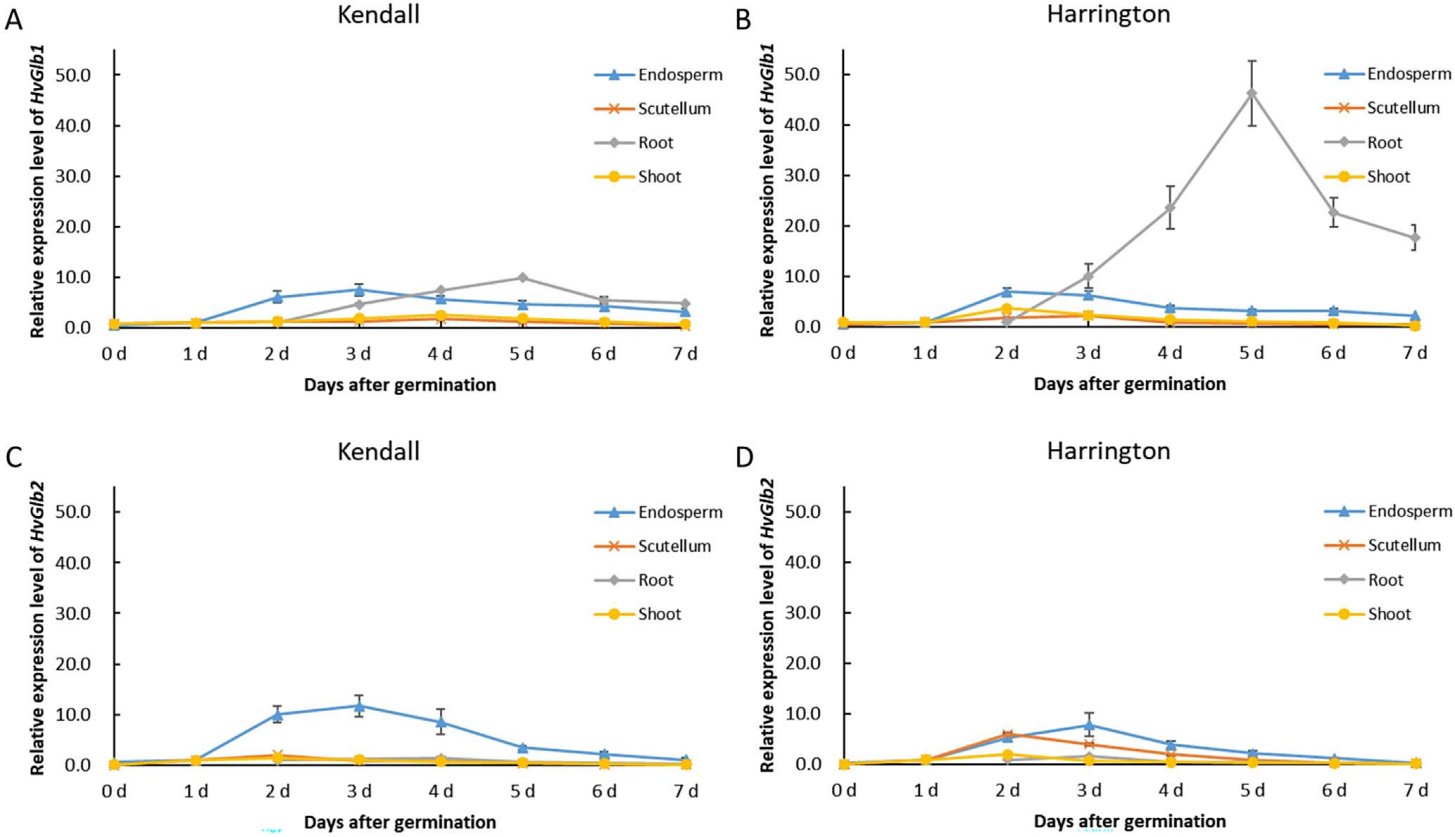
Relative expression level of *HvGlb* genes in four tissues of Kendall and Harrington. (A) Relative expression of *HvGlb1* in Kendall; (B) relative expression of *HvGlb1* in Harrington; (C) relative expression of *HvGlb2* in Kendall; (D) relative expression of *HvGlb2* in Harrington.

The expression levels of *HvGlb* genes exhibited a rapid increase after germination and gradually decreased after reaching peak expression at 2–5 days (Figure 4). The expression levels of *HvGlb1* in the endosperm of both cultivars increased up to a maximum of 7‐fold at 2–3 days (Figure 4A,B). Notably, the expression of *HvGlb1* in root exhibited a remarkable disparity between these two cultivars, with Harrington reaching a maximum of over 45‐fold at 5 days, while Kendall only about 10‐fold (Figure 4A,B). The expression levels of *HvGlb2* in the endosperm exhibited a rapid increase, reaching their maximum at 3 days with an 11.7‐fold increase in Kendall and a 7.9‐fold increase in Harrington (Figure 4C,D). However, the expression level of *HvGlb2* in Harrington scutellum was much higher than that in Kendall scutellum at 2 days.

**FIGURE 5:**
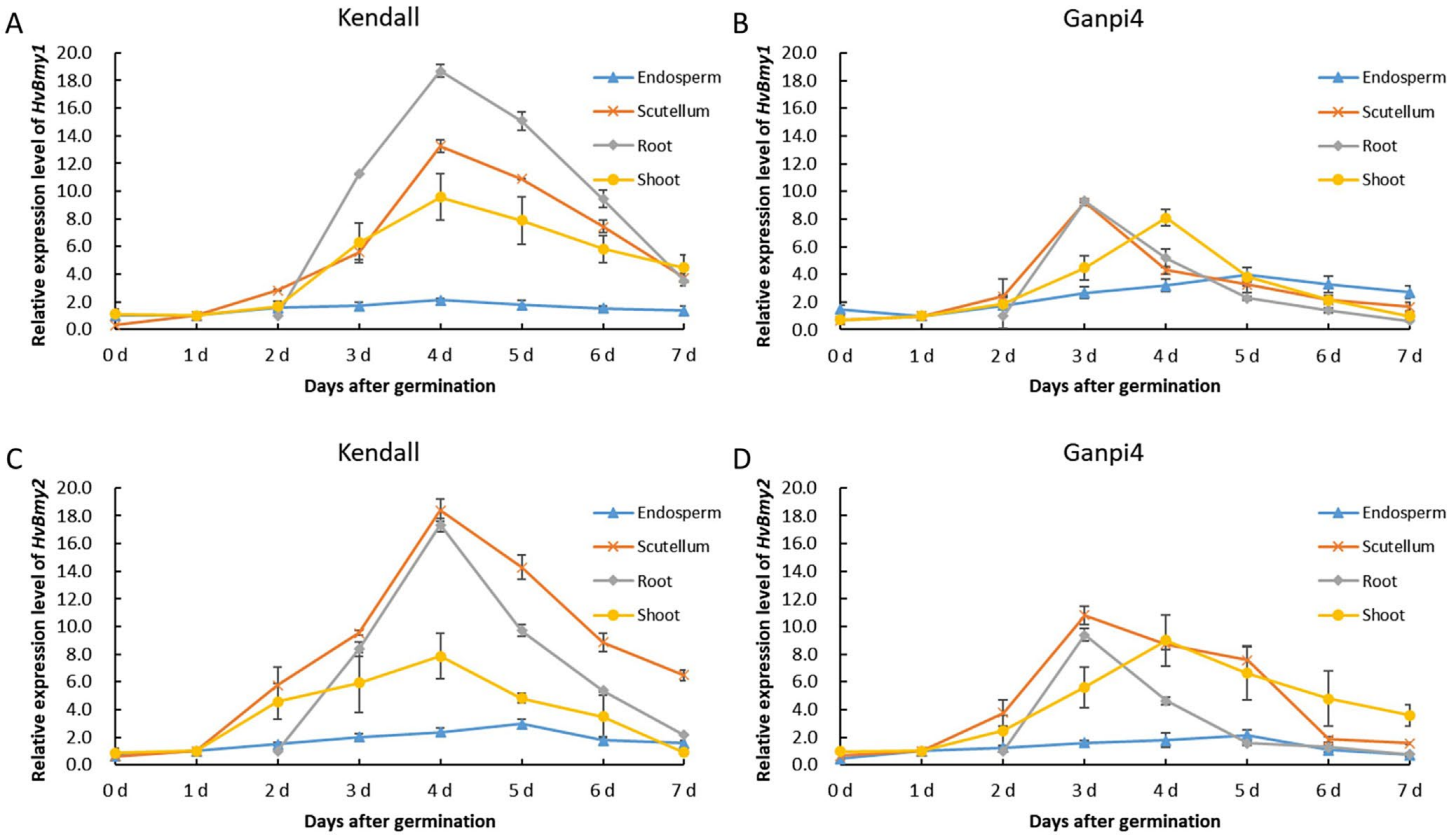
Relative expression level of *HvBmy* genes in four tissues of Kendall and Harrington. (A) Relative expression of *HvBmy1* in Kendall; (B) relative expression of *HvBmy1* in Harrington; (C) relative expression of *HvBmy2* in Kendall; (D) relative expression of *HvBmy2* in Harrington.

The expression levels of *HvBmy* genes in scutellum, root, and shoot exhibited a gradual increase starting from 2 days of germination and reached their peak at 3–4 days (Figure 5). The expression levels in endosperm were much lower than that in other parts. Similar expression patterns were observed for both *HvBmy1* and *HvBmy2*. The expression levels of both genes in root and scutellum were comparatively lower in Ganpi4 than in Kendall, however, Ganpi4 reached its maximum levels 1 day earlier than Kendall (Figure 5).

## 4 Discussion

### 4.1 β‐Amylase Activity and Malting Quality Traits

Malting is one of the most important end uses of barley, and thus improving malting quality has been a primary objective for breeders over the past few decades. However, limited progress has been made due to the insufficient genetic understanding pertaining to malting quality traits. Hydrolytic enzymes are synthesized or activated during malting to degrade endosperm cell wall, facilitating further enzymatic hydrolysis of starch, proteins, and lipids (Bamforth 2009). Amylase is very important to mobilize fermentable sugars from starch (Daba et al. 2019), and β‐amylase is the most important enzyme in terms of DP (Coventry et al. 2003; Duke and Henson 2009; Evans, Li, and Eglinton 2008; Filichkin et al. 2010; Henson and Duke 2008). In this work, Ganpi4 displayed the highest DP reaching 471.27 WK (Figure 1C), and the β‐amylase activity of Ganpi4 consistently exhibited higher levels compared with other cultivars throughout the entire germination period (Figure 2B). In addition, the β‐amylase activity demonstrated a significantly positive correlation with DP when all 14 cultivars were included for correlation analysis (Table 4). These results were in accordance with previous research. The synthesis of β‐amylase occurs in the aleurone layer, and it is subsequently released from a protein complex to become active (Grime and Briggs 1996; Guerin, Lance, and Wallace 1992). There are two forms of β‐amylase, β‐amylase1, and β‐amylase2 (Vinje et al. 2011a, 2011b). It was reported that the majority of β‐amylase activity in barley malt and wort was primarily attributed to β‐amylase1 (Henson and Duke 2016). However, both *HvBmy1* and *HvBmy2* showed a similar expression pattern in this work, suggesting that both genes contributed to DP. Interestingly, Ganpi4, which exhibited the highest β‐amylase activity during germination, demonstrated a relatively lesser increase in the expression levels of both genes compared to Kendall, which had a moderate DP (Figure 5). However, the expression of both genes in Ganpi4 showed a rapid increase in scutellum and root, peaking 1 day earlier than Kendall. This suggested that Ganpi4 might synthesize β‐amylase earlier and accumulate higher levels of it to achieve higher β‐amylase activity, and consequently a higher DP.

### 4.2 α‐Amylase Activity and Malting Quality Traits

α‐amylase is an endohydrolase that facilitates the hydrolysis of internal α‐(1,4)‐glucosyl linkages within amylose and amylopectin molecules, thereby playing a pivotal role in starch degradation (Evans et al. 2005). High α‐amylase activity is consistently associated with elevated levels of fermentable sugars and subsequently, increased MEX, which is a core characteristic of malting quality determining the final output of beer during fermentation (Islamovic et al. 2014). In this work, α‐amylase activity was observed to be significantly and positively correlated with MEX (Table 4). The α‐amylase activity of Kendall and Harrington increased more rapidly and reached a higher peak than other cultivars (Figure 2A). These two cultivars exhibited higher MEX levels than other cultivars, with Kendall in particular reaching an impressive MEX level of 84.33% (Figure 1A; Table 3). Moreover, α‐amylase activity also exhibited significant and positive correlation with FAN and KI, whereas significant but negative correlation with VIS (Table 4). In addition, Kendall and Harrington exhibited similar expression patterns of *HvAmy1* and *HvAmy2* during germination, although the expression levels of *HvAmy1* and *HvAmy2* in Kendall endosperm peaked 1 day earlier than that in Harrington (Figure 3). Large numbers of α‐amylase are synthesized by scutellum epithelial cells and aleurone layer cells and then secreted into endosperm to degrade starch (Fu et al. 2025; Macgregor et al. 1984; Mundy, Brandt, and Fincher 1985; Ranki 1990), thus the expression levels of both *HvAmy1* and *HvAmy2* in endosperm and scutellum were much higher than that in root (Figure 3).

### 4.3 β‐Glucanase Activity and Malting Quality Traits

The endosperm cell wall of barley is primarily composed of BG, with β‐glucanase being the primary enzyme responsible for its degradation (Gianinetti 2009). It was reported that low activity of malt β‐glucanase led to an increase in BG content and a decrease in DP, thus affecting the composition of fermentable sugars in the wort (Rani et al. 2024). Another recent research revealed that lack of β‐glucanase activity resulted in reduced DP, and thereby insufficient starch degradation or fermentation (Kihara et al. 2024). β‐glucanase is encoded by two genes, namely *HvGlb1* and *HvGlb2*, and both enzymes are synthesized in the aleurone (Kuusela et al. 2004; Matthies et al. 2009). The expression of both *HvGlb1* and *HvGlb2* increased to the peak levels at 2–3 days (Figure 4). In root, only *HvGlb1* was highly expressed at a relatively late stage (5 days) with Harrington showing much higher expression level (> 45 times; Figure 4A,B). During germination, the proteinases are responsible for catalyzing the hydrolysis of storage proteins into soluble proteins, peptides, and amino acids (Simpson 2001). High FAN level is crucial for the growth of yeast during fermentation (Islamovic et al. 2014). It was reported that proteinase activity exhibited dramatic variation among barley genotypes and was correlated positively with FAN and KI (Kihara et al. 2002). The present study revealed a significantly positive correlation between the activities of polysaccharide hydrolyzing enzymes (β‐glucanase and α‐amylase) and FAN as well as KI (Table 4). The induced expression of *HvAmy1* and *HvAmy2* in endosperm and scutellum, along with *HvGlb1* and *HvGlb2* in endosperm, led to the increased synthesis of hydrolases (α‐amylase and β‐glucanase) during germination. This resulted in greater endosperm cell wall (primarily consisting of β‐glucan) and protein modification during germination, leading to the high FAN and KI in Kendall and Harrington (Figure 1D,E).

## 5 Conclusion

The present study investigated the relationships between the activities of three saccharide hydrolyzing enzymes and five malting quality traits across 14 malt barley cultivars and explored the expression patterns of six enzyme genes in diverse tissues of cultivars with elite malting quality traits. Overall, α‐amylase activity was significantly correlated with MEX, VIS, FAN, and KI, whereas β‐amylase activity was significantly correlated solely with DP. β‐glucanase activity was significantly correlated with FAN and KI. The expression of genes encoding α‐amylase, β‐amylase, and β‐glucanase were remarkably increased and exhibited similar patterns during germination in cultivars with elite malt quality traits. These results enhance our understanding of the relations between enzyme activity and malting quality traits and may facilitate further breeding for malt barley cultivars.

## Author Contributions

**Kangfeng Cai:** formal analysis (equal), writing – original draft (equal), writing – review and editing (equal). **Xiaojian Wu:** formal analysis (equal), investigation (lead), writing – original draft (equal). **Wenhao Yue:** formal analysis (supporting). **Lei Liu:** formal analysis (supporting). **Xiujuan Song:** formal analysis (supporting). **Fangying Ge:** formal analysis (supporting). **Qiuyu Wang:** formal analysis (supporting). **Junmei Wang:** conceptualization (lead), formal analysis (equal), funding acquisition (lead), project administration (lead), resources (supporting), supervision (lead), writing – review and editing (equal).

## Ethics Statement

The authors have nothing to report.

## Consent

Written informed consent was obtained from all study participants.

## Conflicts of Interest

The authors declare no conflicts of interest.

## Data Availability

The authors have nothing to report.

## References

[fsn34702-bib-0001] Bamforth, C. W. 2009. “Current Perspectives on the Role of Enzymes in Brewing.” Journal of Cereal Science 50, no. 3: 353–357.

[fsn34702-bib-0002] Bamforth, C. W. 2003. “Barley and Malt Starch in Brewing: A General Review.” Technical Quarterly & the MBAA Communicator 2: 40.

[fsn34702-bib-0003] Briggs, D. E. 1998. Malts and Malting. New York: Springer New York.

[fsn34702-bib-0004] Coventry, S. J. , H. M. Collins , A. R. Barr , et al. 2003. “Use of Putative QTLs and Structural Genes in Marker Assisted Selection for Diastatic Power in Malting Barley ( *Hordeum vulgare* L.).” Australian Journal of Agricultural Research 54, no. 11–12: 1241–1250.

[fsn34702-bib-0005] Cu, S. T. , T. J. March , S. Stewart , et al. 2016. “Genetic Analysis of Grain and Malt Quality in an Elite Barley Population.” Molecular Breeding 36, no. 9: 1–16.

[fsn34702-bib-0006] Daba, S. , R. Horsley , P. Schwarz , S. Chao , F. Capettini , and M. Mohammadi . 2019. “Association and Genome Analyses to Propose Putative Candidate Genes for Malt Quality Traits.” Journal of the Science of Food and Agriculture 99, no. 6: 2775–2785. 30430569 10.1002/jsfa.9485

[fsn34702-bib-0007] Duke, S. H. , and C. A. Henson . 2009. “A Comparison of Barley Malt Osmolyte Concentrations and Standard Malt Quality Measurements as Indicators of Barley Malt Amylolytic Enzyme Activities.” Journal of the American Society of Brewing Chemists 67, no. 4: 206–216.

[fsn34702-bib-0008] Evans, D. E. , H. Collins , J. Eglinton , and A. Wilhelmson . 2005. “Assessing the Impact of the Level of Diastatic Power Enzymes and Their Thermostability on the Hydrolysis of Starch During Wort Production to Predict Malt Fermentability.” Journal of the American Society of Brewing Chemists 63, no. 4: 185–198.

[fsn34702-bib-0009] Evans, D. E. , C. Li , and J. K. Eglinton . 2008. “Improved Prediction of Malt Fermentability by Measurement of the Diastatic Power Enzymes β‐Amylase, α‐Amylase, and Limit Dextrinase: I. Survey of the Levels of Diastatic Power Enzymes in Commercial Malts.” Journal of the American Society of Brewing Chemists 66: 223–232.

[fsn34702-bib-0010] Filichkin, T. P. , M. A. Vinje , A. D. Budde , et al. 2010. “Phenotypic Variation for Diastatic Power, β‐Amylase Activity, and β‐Amylase Thermostability vs. Allelic Variation at the *Bmy1* Locus in a Sample of North American Barley Germplasm.” Crop Science 50, no. 3: 826–834.

[fsn34702-bib-0011] Fox, G. P. , J. F. Panozzo , C. D. Li , R. C. M. Lance , P. A. Inkerman , and R. J. Henry . 2003. “Molecular Basis of Barley Quality.” Australian Journal of Agricultural Research 54: 1081–1101.

[fsn34702-bib-0012] Fu, D. , W. Wenhua , G. Mustafa , Y. Yang , and P. Yang . 2025. “Molecular Mechanisms of Rice Seed Germination.” New Crops 2: 100051.

[fsn34702-bib-0013] Georg‐Kraemer, J. E. , E. C. Mundstock , and S. Cavalli‐Molina . 2001. “Developmental Expression of Amylases During Barley Malting.” Journal of Cereal Science 33, no. 3: 279–288.

[fsn34702-bib-0014] Gianinetti, A. 2009. “A Theoretical Framework for β‐Glucan Degradation During Barley Malting.” Theory in Biosciences 128, no. 2: 97–108. 19130112 10.1007/s12064-008-0055-7

[fsn34702-bib-0015] Grime, K. H. , and D. E. Briggs . 1996. “The Release of Bound β‐Amylase by Macromolecules.” Journal of the Institute of Brewing 102, no. 4: 261–270.

[fsn34702-bib-0016] Guerin, J. R. , R. C. M. Lance , and W. Wallace . 1992. “Release and Activation of Barley Beta‐Amylase by Malt Endopeptidases.” Journal of Cereal Science 15, no. 1: 5–14.

[fsn34702-bib-0017] Henson, C. A. , and S. H. Duke . 2008. “A Comparison of Standard and Nonstandard Measures of Malt Quality.” Journal of the American Society of Brewing Chemists 66, no. 1: 11–19.

[fsn34702-bib-0018] Henson, C. A. , and S. H. Duke . 2016. “Maltose Effects on Barley Malt Diastatic Power Enzyme Activity and Thermostability at High Isothermal Mashing Temperatures: I. β‐Amylase.” Journal of the American Society of Brewing Chemists 74: 100–112.

[fsn34702-bib-0019] Islamovic, E. , D. E. Obert , A. D. Budde , et al. 2014. “Quantitative Trait Loci of Barley Malting Quality Trait Components in the Stellar/01Ab8219 Mapping Population.” Molecular Breeding 34, no. 1: 59–73.

[fsn34702-bib-0020] Kihara, M. , Y. Kozaki , K. Takoi , C. Shimizu , K. Ogushi , and T. Hoki . 2024. “Malting and Brewing Performance of β‐Amylase‐Deficient Barley.” Journal of the American Society of Brewing Chemists 82, no. 4: 317–322.

[fsn34702-bib-0021] Kihara, M. , W. Saito , Y. Okada , T. Kaneko , T. Asakura , and K. Ito . 2002. “Relationship Between Proteinase Activity During Malting and Malt Quality.” Journal of the Institute of Brewing 108, no. 3: 371–376.

[fsn34702-bib-0022] Kumar, V. , S. K. Chaturvedi , and G. P. Singh . 2023. “Brief Review of Malting Quality and Frontier Areas in Barley.” Cereal Research Communications 51, no. 1: 45–59.

[fsn34702-bib-0023] Kuusela, P. , J. J. Hämäläinen , P. Reinikainen , and J. Olkku . 2004. “A Simulation Model for the Control of Beta‐Glucanase Activity and Beta‐Glucan Degradation During Germination in Malting.” Journal of the Institute of Brewing 110, no. 4: 309–319.

[fsn34702-bib-0024] Lai, J. Y. , A. Speers , A. T. Paulson , and R. J. Stewart . 2004. “Effects of β‐Glucans and Environmental Factors on the Viscosities of Wort and Beer.” Journal of the Institute of Brewing 110, no. 2: 104–116.

[fsn34702-bib-0025] Laitila, A. , E. Kotaviita , P. Peltola , S. Home , and A. Wilhelmson . 2007. “Indigenous Microbial Community of Barley Greatly Influences Grain Germination and Malt Quality.” Journal of the Institute of Brewing 113, no. 1: 9–20.

[fsn34702-bib-0026] Leisova‐Svobodova, L. , V. Psota , M. Zavrelova , M. Kriz , P. Marik , and Z. Nesvadba . 2024. “Malting Quality Molecular Markers for Barley Breeding.” Czech Journal of Genetics and Plant Breeding 60, no. 2: 70–78.

[fsn34702-bib-0027] Liu, J. , B. Chu , X. Yang , and Y. Jin . 2021. “Relationship Between the Index of Protein Modification (Kolbach Index) and Degradation of Macromolecules in Wheat Malt.” Journal of Food Science 86, no. 6: 2300–2311. 33929729 10.1111/1750-3841.15701

[fsn34702-bib-0028] Macgregor, A. W. , F. Helen Macdougall , C. Mayer , and J. Daussant . 1984. “Changes in Levels of α‐Amylase Components in Barley Tissues During Germination and Early Seedling Growth.” Plant Physiology 75: 203–206. 16663571 10.1104/pp.75.1.203 PMC1066862

[fsn34702-bib-0029] Matthies, I. E. , S. Weise , J. Förster , and M. S. Röder . 2009. “Association Mapping and Marker Development of the Candidate Genes (1 → 3),(1 → 4)‐β‐d‐Glucan‐4‐Glucanohydrolase and (1 → 4)‐β‐Xylan‐Endohydrolase 1 for Malting Quality in Barley.” Euphytica 170, no. 1: 109–122.

[fsn34702-bib-0030] Mundy, J. , A. Brandt , and G. B. Fincher . 1985. “Messenger RNAs From the Scutellum and Aleurone of Germinating Barley Encode (1→3,1→4)‐β‐D‐Glucanase, α‐Amylase and Carboxypeptidase.” Plant Physiology 79, no. 3: 867–871. 16664507 10.1104/pp.79.3.867 PMC1074986

[fsn34702-bib-0031] Qi, J.‐c. , J.‐x. Chen , J.‐m. Wang , W. Fei‐bo , L.‐p. Cao , and G.‐p. Zhang . 2005. “Protein and Hordein Fraction Content in Barley Seeds as Affected by Sowing Date and Their Relations to Malting Quality.” Journal of Zhejiang University‐Science B 6, no. 11: 1069–1075. 16252340 10.1631/jzus.2005.B1069 PMC1390653

[fsn34702-bib-0032] Rani, H. , R. D. Bhardwaj , R. Sen , et al. 2024. “Deciphering the Potential of Diverse Barley Genotypes for Improving the Malt Quality.” Journal of Stored Products Research 105: 102247.

[fsn34702-bib-0033] Rani, H. , and S. J. Whitcomb . 2025. “Integrative LC‐MS and GC‐MS Metabolic Profiling Unveils Dynamic Changes During Barley Malting.” Food Chemistry 463: 141480. 39426241 10.1016/j.foodchem.2024.141480

[fsn34702-bib-0034] Ranki, H. 1990. “Secretion of α‐Amylase by the Epithelium of Barley Scutellum.” Journal of the Institute of Brewing 96, no. 5: 307–309.

[fsn34702-bib-0035] Simpson, D. J. 2001. “Proteolytic Degradation of Cereal Prolamins—The Problem With Proline.” Plant Science 161, no. 5: 825–838.

[fsn34702-bib-0036] Vinje, M. A. , D. K. Willis , S. H. Duke , and C. A. Henson . 2011a. “Differential Expression of Two β‐Amylase Genes (*Bmy1* and *Bmy2*) in Developing and Mature Barley Grain.” Planta 233, no. 5: 1001–1010. 21279650 10.1007/s00425-011-1348-5

[fsn34702-bib-0037] Vinje, M. A. , D. K. Willis , S. H. Duke , and C. A. Henson . 2011b. “Differential RNA Expression of *Bmy1* During Barley Seed Development and the Association With β‐Amylase Accumulation, Activity, and Total Protein.” Plant Physiology and Biochemistry 49, no. 1: 39–45. 20974538 10.1016/j.plaphy.2010.09.019

[fsn34702-bib-0038] Wang, J. , J. Yang , W. Hua , et al. 2018. “QTL Mapping Reveals the Relationship Between Pasting Properties and Malt Extract in Barley.” International Journal of Molecular Sciences 19, no. 11: 6–8. 10.3390/ijms19113559 PMC6275068 30424480

[fsn34702-bib-0039] Wang, J. , J. Yang , Q. Zhang , et al. 2015. “Mapping a Major QTL for Malt Extract of Barley From a Cross Between TX9425 × Naso Nijo.” Theoretical and Applied Genetics 128, no. 5: 943–952. 25773294 10.1007/s00122-015-2481-5

[fsn34702-bib-0040] Yousif, A. M. , and D. Evan Evans . 2020. “Changes in Malt Quality During Production in Two Commercial Malt Houses.” Journal of the Institute of Brewing 126, no. 3: 233–252.

